# Comparison of semi-continuous and interrupted suture technique for aortic valve replacement

**DOI:** 10.1186/1749-8090-8-S2-O1

**Published:** 2013-10-23

**Authors:** Q Chen, C Di-Salvo, N Roberts, J Yap

**Affiliations:** 1 Cardiac Surgery, The Heart Hospital, London, UK

## Background

Previous study suggested semi continuous technique increases the risk of re-operation for paraprosthetic leak AVR compared with interrupted suture technique. We aim to evaluate our experience in these two techniques performed in our institution.

## Methods

Retrospective review of patients who underwent aortic valve replacement (AVR) with or without combined cardiac procedures from April 2002 to September 2012. The data was collected from hospital electronic data base and medical notes.

## Results

The number of patients underwent AVR including combined cardiac procedures was 1005 with semi continuous technique (group 1) and 1110 with interrupted technique (group 2). The hospital mortality was 4.68% in group 1 and 3.96% in group 2. Cardiopulmonary bypass (CPB) time and aortic cross clamp (XC) time for isolated AVR was 89 ± 42 (41 – 379) and 63 ± 31 (29 – 258) in group 1, 98 ± 34 ( 44 – 377) and 72 ± 26 (30 – 236) in group 2, p=0.0002, p<0.0001, respectively. Late cardiac reoperation was performed in 23 patients (2.3%) in group 1 and in 24 patients (2.2%) in group 2. Late repeat operation for AVR was performed in 5 patients (0.5%) in group 1, and in 5 patients (0.45%) in group 2. There were 51 late deaths (10.9%) group 1 and 48 late deaths (9.2%) in group 2.

## Conclusions

The incidence for repeat operation for AVR following primary AVR was low. There was no significant difference in terms of hospital mortality, repeat operation for AVR, and late survival between groups of patients with semi continuous and interrupted suture techniques. Semi continuous suture technique, however, reduces the CPB and aortic XC time compared with interrupted suture technique.

